# DNA Immunization for HIV Vaccine Development

**DOI:** 10.3390/vaccines2010138

**Published:** 2014-02-25

**Authors:** Yuxin Chen, Shixia Wang, Shan Lu

**Affiliations:** Department of Medicine, University of Massachusetts Medical School, Worcester, MA 01605, USA; E-Mail: shixia.wang@umassmed.edu

**Keywords:** HIV-1, AIDS, vaccine, DNA immunization

## Abstract

DNA vaccination has been studied in the last 20 years for HIV vaccine research. Significant experience has been accumulated in vector design, antigen optimization, delivery approaches and the use of DNA immunization as part of a prime-boost HIV vaccination strategy. Key historical data and future outlook are presented. With better understanding on the potential of DNA immunization and recent progress in HIV vaccine research, it is anticipated that DNA immunization will play a more significant role in the future of HIV vaccine development.

## 1. Introduction

More than 20 years have passed since the introduction of the concept of the DNA vaccine when several groups of scientists independently reported the use of this novel technology to elicit immune responses in small animal models against either a marker protein [1] or various model viral antigens [2,3,4,5,6,7]. The HIV-1 DNA vaccine was not only among this first group of initial reports [2,3], but was also one of the first DNA vaccines tested in non-human primates [8,9,10,11] and the first tested in humans [12,13,14]. 

One reason for the excitement towards the discovery of DNA vaccination was the potential of DNA vaccines to elicit T cell-mediated immunity. In the last several decades, the role of T cells in protective immunity has been increasingly realized by basic immunologists yet it was also frustrating to see limited progress in eliciting T cell immune responses by vaccination, especially with the use of traditional vaccines. Inactivated, subunit, or recombinant protein vaccines, which represent the majority of licensed human vaccines, are known to be poorly immunogenic towards the elicitation of T cell immune responses, especially CD8^+^ T cell responses. In theory, live attenuated vaccines are capable of eliciting high quality T cell immune responses but given safety concerns associated with a modified live pathogen, the selection of this form of vaccine has been declining since the middle of the 20th century.

The introduction of DNA vaccination in the early 1990s provided a completely new opportunity for novel vaccine efforts in inducing T cell immune responses [15]. Given the challenge of developing an effective HIV vaccine and the understanding that cell-mediated immunity (CMI) is critical in the control of infection in HIV-1 positive patients [16,17], the HIV vaccine field welcomed the DNA vaccine concept and has contributed to the many further advancements of DNA vaccine technology.

This review will focus on various optimizations of DNA vaccine technology from the last 20 years including results from key representative studies. More importantly, a vision will be presented on the transition from using DNA immunization, mainly for the induction of T cells, to the promising future of using this technology to elicit high quality antibody responses in the post-RV144 HIV vaccine landscape.

### 1.1. Adjuvanted HIV-1 DNA Vaccines

The first human DNA vaccine study was done in HIV-1 infected patients as a Phase I clinical trial for safety analysis, followed by studies in healthy, HIV-1 negative volunteers [12,13]. These studies confirmed the overall safety profile for DNA vaccines but the immunogenicity results were disappointing. The magnitude and frequency of T cell immune responses, as the main objectives in these early clinical trials, were low; HIV-1 antigen-specific antibody responses were generally low or below detection.

In order to improve the immunogenicity of DNA vaccines in humans, one key strategy, which is still part of many current clinical HIV-1 DNA vaccine formulations, is the use of novel “molecular adjuvants” [18,19]. Unlike traditional adjuvants, which are usually chemical compounds formulated with protein-based vaccines, molecular adjuvants, similar to DNA vaccines, are DNA plasmid-based expression vectors with gene inserts coding for various cytokines that can stimulate the host immune system. One unique advantage of molecular adjuvants is that they can be co-delivered with DNA vaccine plasmids without any extra formulation work. They can transduce cells at the site of DNA vaccine inoculation, and express cytokines to serve as adjuvants for DNA vaccines [19]. 

Traditional adjuvants elicit a broad spectrum of immune responses while gene-based adjuvants, in general, express only one particular cytokine that focuses on a key immune regulatory pathway. Based on the profile of immune responses to be elicited, molecular adjuvants can be grouped as Th1 or Th2 adjuvants. Th1 adjuvants include DNA plasmids expressing IL-2, IFN-γ, IL-12, and IL-15, which are used mainly to augment cellular immunity. For example, in clinical trial HVTN044, when IL-2 was delivered in the form of a DNA plasmid coding for the IL-2 fusion protein along with HIV vaccines, enhanced T cell responses were observed [20]. Molecular adjuvant IL-12 was able to increase CD8^+^ T cell responses in mice [21], control viremia, and improve clinical outcome following SHIV challenge in a non-human primate model [22]. IL-15, unlike most Th1 adjuvants, showed a balanced effect in augmenting both cellular and humoral immunities. This makes IL-15 an attractive adjuvant to be included in HIV-1 DNA vaccines. However, there was no significant increase in the response rate with the addition of IL-12 or IL-15 in a recently completed clinical trial HVTN070 [23]. 

Other molecular adjuvants express Th2 cytokines, such as GM-CSF, IL-1, and IL-4, and are used to enhance humoral immunity [19]. DNA vaccines, when used in combination with GM-CSF, improved antigen-specific antibody responses and enhanced lymphoproliferation in mice [24]. In rhesus macaques, co-delivery of GM-CSF-expressing plasmid and SIV DNA vaccines followed by a MVA boost resulted in enhanced binding antibody and neutralizing antibody responses, and increased protection against SIV challenge [25]. Whether Th2 molecular adjuvants are able to improve the immunogenicity of HIV-1 DNA vaccines in human is under active clinical investigation. 

### 1.2. Optimization of DNA Vaccine Vector Designs

Over the past two decades, significant technical improvements to the design of DNA vaccine vectors have contributed to much improved antigen expression and immunogenicity of HIV-1 DNA vaccines in both animal and human studies.

First, codon optimization of immunogen genes [26,27,28,29,30] was found highly effective in elevating immunogen protein expression as shown by *in vitro* experiments, and enhancing T cell and antibody responses *in vivo*. Because HIV-1 uses a codon preference that is significantly different from that used in mammalian cells, it was demonstrated that modified codon usage matching that used in mammalian cells, without changing the coding amino acid sequences, can better match the tRNAs preferentially used in mammalian cells and, thus, enhance the protein expression of DNA vaccine immunogens [31].

Second, replacing the HIV-1 Env leader sequence with signal peptides from other mammalian proteins was able to greatly improve the protein expression of Env-based HIV-1 DNA vaccines; however, more than one mechanism may be involved in this improved production of Env immunogens by DNA vaccines. Because the main purpose of Env in an HIV-1 vaccine design is to elicit protective antibody responses, certain mammalian leaders that can produce a larger quantity of secreted soluble Env proteins, compared with the original Env leader, will lead to higher antibody response levels. It is also likely that the highly unusual and defective nature of the original HIV-1’s Env leader sequence, *i.e.*, multiple charged amino acid residues, may be responsible for the overall low level production of Env proteins and limited secretion of Env out of cells. As shown by early efforts in improving the production of Env recombinant proteins, the leader sequence of the human tissue plasminogen activator (tPA) was able to greatly improve the expression and secretion of HIV-1 by the mammalian cell expression system [32,33]. When the same tPA leader was used for Env DNA vaccines, higher *in vitro* Env immunogen expression and *in vivo* Env-specific antibody responses were observed in mice [34].

Third, viral promoter efficiency plays a critical role in improving gene transcription of HIV-1 DNA vaccines. In early studies, strong promoters were derived from certain human oncogenic viruses, including LTR from Rous sarcoma virus and the SV40 early promoter [35]. However, in the last 20 years, intermediate-early gene 1 promoter adopted from human cytomegalovirus (HCMV), a non-carcinogenic virus, has been widely used with high efficiency in most DNA vaccine designs including HIV-1 DNA vaccines [4]. Furthermore, adding an intron A sequence from HCMV to an immediate downstream region of the HCMV promoter was able to further enhance the immunogenicity of HIV-1 DNA vaccines [32]. The other important promoter used for HIV-1 DNA vaccines is the CMV enhancer/promoter with the HTLV-1 R region (CMV/R), the regulatory R region from the 5' long terminal repeat (LTR) of human T cell leukemia virus type 1 (HTLV-1). As a transcriptional and posttranscriptional enhancer, this additional R region substantially enhanced transgene expression by 5 to 10 fold and further improved the cellular immune response [36]. 

Not only are the above three key elements of vector design individually important for a highly immunogenic HIV-1 DNA vaccine, they can also be combined in the same DNA vaccine to achieve a synergistic effect for immunogen expression and immunogenicity of HIV-1 Env DNA vaccines [34,37].

There are additional DNA vaccine designs that have shown various levels of enhancing effects on HIV-1 DNA vaccines, such as the use of a C3d sequence at the *C*-terminus of the HIV-1 Env insert [38]. It was thought that C3d could help elicit antibody undergoing more rapid avidity maturation [39]. However, inclusion of C3d did not demonstrate a synergistic effect in a codon-optimized Env DNA vaccine [40] and later was not included in HIV DNA vaccines.

### 1.3. Antigen Engineering

Two failed phase III HIV-1 vaccine trials using recombinant gp120 proteins (AIDSVAX) [41,42,43] led to the conclusion in the HIV vaccine field that a monomeric gp120 immunogen may not be effective in eliciting protective immune responses. Designing more effective HIV-1 Env immunogens is a major challenge for the development of next generation HIV-1 vaccines. The DNA vaccine approach has become a highly useful tool in this line of work.

Because gp160, the full length form of the HIV-1 Env glycoprotein, is membrane-anchored and hard to be expressed as a secreted protein, most recombinant protein-based HIV-1 Env vaccines use the gp120 or gp140 forms, which include only the extracellular portion of the Env protein and, therefore, neither form is in its natural trimer status. The DNA vaccination approach offers a unique opportunity to conduct a direct comparison among gp120, gp140, and gp160 forms of Env immunogens.

When gp120, gp140, and gp160 DNA vaccines were compared directly in a rabbit study, gp120 was the most immunogenic form in eliciting antibodies against Env protein, gp140 was less effective, and gp160 was the least immunogenic [44]. Presumably, gp160 is not secreted *in vivo*, possibly reducing its immunogenicity as evidenced by lower levels of binding and functional antibodies compared with sera elicited by gp120 and gp140 DNA vaccines. In this study, gp140 with an intact cleavage site between gp120 and gp41 was used; however, gp140 can also be designed with the natural cleavage site mutated as a non-cleavable gp140. When DNA vaccines expressing either cleavable or non-cleavable gp140 forms were compared for their immunogenicity in rabbits, the non-cleavable gp140 was somewhat more immunogenic in eliciting binding antibodies than the cleavable gp140, but the cleavable gp140 (the natural form of gp140) was more effective in eliciting neutralizing antibodies, indicating the cleavable gp140 may be more effective in preserving conformational and functional epitopes [45], which was supported by results from non-DNA HIV Env design studies [46].

While we remain mindful of the non-trimer nature of gp120 immunogens, there is no direct DNA vaccine immunogenicity comparison data that has shown significant benefits to using other forms of Env designs to elicit a better quality of antibodies, such as neutralizing antibody activities against Tier 2 more resistant viral isolates. 

DNA vaccines have been used to test Env immunogens with modified sequences. One well-studied DNA vaccine with a modified immunogen is a gp145 DNA vaccine developed by the Vaccine Research Center at US NIH [47]. In this design, multiple deletions at the cleavage site, at the fusogenic domain, and in the interspace between heptad repeats 1 and 2 were introduced based on the idea that it can stabilize and expose the functional domain of the protein that is present in an extended helical structure [47]. The original data from this mice study showed that it failed to generate consistently high level antibody responses. In phase 1 clinical studies VRC 009 and VRC 010, gp145 DNA vaccines were used as the priming immunization followed by an Ad5-based viral vector vaccine boost [48]. High ELISA binding titers were identified in human immune sera against the autologous gp145 immunogens but no data on its recognition of natural Env proteins (gp120 or other forms) was provided. Only 28% of vaccinees generated low levels of serum neutralization activity against two most sensitive viruses, SF162 and MW965.26. No neutralizing activities were detected against the other selected primary virus isolates, raising the possibility that modified Env immunogens with multiple deletions may have negatively impacted the integrity of Env immunogens and the resulting immune sera may not recognize the original Env immunogens.

DNA vaccines were also used to test other novel Env immunogen designs, such as Env immunogens with consensus sequences [49]. However, it is frequently ignored that Env consensus sequences only cover constant regions; variable regions of Env are not part of the consensus sequence because the variable region sequences are too variable to reach a consensus. Instead, the natural sequences of the variable regions from certain HIV-1 isolates were used to combine with the consensus sequences of constant regions to form final full length Env sequences [50]. However, no broadly neutralizing antibodies were elicited with such consensus Env immunogens.

Antigen engineering is also important for DNA vaccines expressing HIV-1 antigens other than Env. For example, the immunogenicity and type of immune response elicited by a wild type Gag DNA vaccine are quite different from another Gag DNA vaccine that includes an additional tPA leader sequence [51]. DNA vaccines encoding full length wild type Gag expressed an intracellular Gag antigen and generated high level Gag-specific T cell responses whereas adding a tPA leader sequence led to a secreted Gag protein along with a greatly enhanced Gag-specific antibody response but decreased Gag-specific T cell responses compared to the wild type Gag DNA vaccine design.

### 1.4. Polyvalent and Multigene Formulations

One of the unique technical advantages for DNA vaccines is the possibility of delivering more than one DNA vaccine components at the same time. The daunting sequence diversity of HIV-1 is a key challenge to any candidate vaccines to elicit the broad antibody and cellular immune responses. While it is highly desired, there is limited progress in designing an immunogen that can effectively elicit a broad neutralizing antibody response. An alternative strategy, which has been adopted in many licensed human vaccines, is the polyvalent formulation including similar immunogens from a given pathogen with different immunological subtypes [52]. The difficulty of applying this strategy to HIV vaccine development is the fact that there are no clear serotypes or immunotypes based on HIV-1 Env immunogen. However, a pilot study using the DNA prime-protein boost approach in rabbits to compare the immunogenicity between monovalent and polyvalent gp120 formulations expressing primary Env antigens from several major subtypes of circulating HIV-1 viral isolates provided promising data to support the use of polyvalent Env formulations as part of the overall HIV vaccine development effort [53]. At the peak level of post final boost immunization, rabbit immune sera elicited by the polyvalent gp120 formulation neutralized 67.86% of a multiclade panel of tested viruses, while the monovalent vaccine elicited sera neutralized only 38.39% viruses. More strikingly, for non-clade B viruses, the polyvalent sera neutralized 56.25% of viruses from clade A, C, D, and E; while the monovalent group had a frequency of only 14.06% neutralization capability. 

A similar idea but requiring additional molecular modifications is the use of “designer’s immunogen” genes encoding several “mosaic” domains, assembled from fragments of natural HIV-1 sequences via a computational optimization method [54]. This approach may be more effective to elicit broad T cell immune responses because mosaic immunogens maximize the coverage of potential T-cell epitopes (peptides of 9 aa length) for a viral population. This approach not only greatly increases the coverage of viral diversity compared to natural sequence vaccine candidates, but also elicits enhanced breadth and depth of epitope recognition of variant sequences of CD8^+^ T lymphocyte epitopes. In mice, a three-set mosaic Env antigens delivered by DNA vaccines elicited increased breadth of CD8^+^ T cell epitopes compared to a DNA vaccine encoding natural Env immunogens [55]. Another study used polyvalent mosaic immunogens derived by *in silico* recombination of natural strains of HIV-1. Rhesus monkeys immunized with this type of mosaic DNA prime and recombinant vaccinia virus boost vaccine regimen elicited increased breadth and depth of epitope recognition of CD8^+^ T cell responses, compared to consensus immunogen [56].

### 1.5. Chemical *vs*. Physical Delivery Approaches

DNA vaccines, when first introduced in the early 1990s, were mainly delivered by simple intramuscular needle injection in small animal models. However, DNA immunization using conventional needle delivery appeared less immunogenic in non-human primates and humans. With recent advancements in DNA vaccine delivery technologies, DNA plasmid delivery methods can be grouped into two major categories: chemical delivery approaches and physical delivery approaches. 

For chemical delivery approaches, DNA plasmids are dissolved in various solutions, with or without polymer carriers (such as lipid, biopolymer, or other chemical compounds), and are delivered by conventional intramuscular or intradermal needle injections, transdermal patches, or through direct mucosal administration. The common feature of such deliveries is that DNA molecules are dissolved in chemical solution and cells at targeted tissues take up DNA plasmids by a low efficiency system.

For the physical delivery approaches, the delivery of DNA vaccines utilizes various physical forces, such as shock wave, high pressure gas, and electrical pulse. In general, the physical approaches require special devices that can produce the external force. The most well known examples are gene gun or an electroporation device. Due to the use of such physical forces, DNA molecules are more likely to be delivered directly inside the cells of the target tissue so the efficiency of DNA vaccines is improved.

The gene gun, one of the early physical delivery approaches, uses high pressure gas to deliver DNA plasmid-coated gold particles into the epidermal layer of the skin, an area rich with antigen presenting cells. Only a small amount of DNA is needed for this approach. The limited dose of DNA vaccines that can be delivered per shot requires multiple shots at each immunization. The gene gun was the first DNA vaccine delivery approach that showed balanced humoral and T cell immune responses in humans, including positive antibody responses at protection levels against hepatitis B surface antigens [57,58]. Using a similar approach, DNA vaccines expressing the HA antigen of seasonal influenza viruses were able to elicit protective levels of hemagglutinin inhibition (HI) antibody responses [59]. There is limited information on the use of the gene gun to deliver HIV-1 DNA vaccines in humans because the gene gun device has been under the control of various pharmaceutical companies and not available for broad human clinical studies.

Biojector, a high pressure, needle-free device, that shoots the DNA plasmid solution into the skin, also provides improved DNA vaccine delivery by directly transfecting DNA into the cells of targeted skin tissue [60]. This device was compared to traditional needle delivery in a phase I clinical trial [61] in the context of DNA prime followed by rAd5 boost immunizations. This study revealed a higher response rate and 3-fold higher magnitude of T cell responses for Biojector delivery compared to the traditonal needle delivery approach.

The electroporation (EP) approach involves delivery of short electrical pulses after needle injection of DNA vaccines. These pulses serve to increase DNA uptake by cells, leading to increased antigen expression and immunogenicity. EP delivery of AIDSVAX, a multi-gene HIV-1 DNA vaccine candidate, increased the magnitude of HIV-1-specific cell-mediated immunity by up to 70-fold over intramuscular injection, as measured by T cell γ-IFN response in humans [62]. A more recent study also confirmed the potency of the electroporation delivery method; the HVTN080 trial showed that 3 times PENNVAX-B (3 mg) delivered via EP elicited a 39% higher positive intracellular cytokine staining (ICS) response rate compared to three vaccinations with PENNVAX-B (6 mg) administered by standard intramuscular injection [23]. One weakness for the EP approach is the high dose of DNA plasmids required for such delivery and the need to include both needle injection and electric shock by an EP device. Furthermore, molecular adjuvant was still included in the above DNA vaccine formulation despite the use of EP delivery, making the whole formulation/delivery package very complicated and potentially expensive.

The relative immunogenicity of traditional needle injection, gene gun, and electroporation was compared in a mouse model using HA antigen of avian influenza subtype H5N1 [63]. Both gene gun and electroporation methods were found more immunogenic than traditional needle injection. Interestingly, electroporation and needle injection elicited Th-1 biased antibody responses whereas gene gun induced a Th-2 dominated antibody response. These findings provide valuable information for further selection of DNA vaccine delivery methods for human applications.

### 1.6. DNA Prime—Viral Vector Boost to Induce HIV-1-Specific T Cell Responses

Since effective T cell immune responses have been correlated with the control of acute viremia in infected subjects, it was attractive to develop an HIV-1 vaccine that is able to induce a robust T cell response. Although early study results showed that HIV-1 DNA vaccines were able to effectively elicit both CD4^+^ and CD8^+^ T cells responses in small animal and non-human primate models [2,3,64,65,66], the immunogenicity of DNA vaccines was poor when used alone in humans. The progress of viral vector-based vaccines has stimulated the idea of combining DNA and viral vector vaccines in a prime-boost format. The common feature of DNA and viral vector vaccines, both being gene-based vaccines, is their abilities to present endogenous antigens to stimulate T cell immune responses. DNA vaccine has been matched with several well developed viral vector vaccines to maximize HIV-1-specific T cell immune responses.

## 2. DNA Prime—Adenoviral Vector Boost Vaccines

One highly potent viral vector vaccine for generating cellular immunity in humans is the adenovirus platform. The most widely used is the Ad5 viral vector. Two human studies comparing the induction of HIV-1-specific CTL responses generated by DNA plasmid vaccines or recombinant serotype 5 adenoviral vector (Ad5) vaccines showed the plasmid DNA was four times less potent in magnitude and response rate than Ad5 vaccines that contained similar HIV antigen cassettes. Further, the concept of DNA prime-Ad5 boost regimen was tested in the non-human primate (NHP) model, the most effective T cell responses were elicited by either Ad5 vector used alone or as a booster after DNA priming compared to DNA or MVA vector alone [67]. Similar results was replicated in a phase I clinical trial, the comparative analysis showed that Gag-specific T cell responses elicited by the DNA prime-Ad5 boost vaccine measured by ELISpot were similar to Ad5/Ad5, but higher than observed with the DNA/DNA regimen [68].

However, the wisdom of using the Ad5 vector is now being challenged following several clinical trials showing disappointing results. The STEP trial tested the replication defective MRKAd5 vector to deliver a gag/pol/nef vaccine that did not provide any protection in a phase IIb trial and instead, may have caused increased viral acquisition in men with pre-existing Ad5 immunity and in uncircumcised men [69]. More recently, the HVTN505 trial, which used a DNA prime-Ad5 boost immunization regimen, also failed to show any protection with this regimen and had higher numbers of HIV-1 infection in vaccine recipients than the placebo recipients [70] although Ad5 seropositive and uncircumcised men were excluded from the HVTN505 trial. While more recent analysis may not show a statistical significance to confirm the risk of using Ad5 vector, it is difficult to anticipate the wide use of the Ad5 vector for HIV-1 vaccine studies in the near future, and future studies may need to use other serotypes of adenoviral vectors that target different cell receptors. 

## 3. DNA Prime—Pox Viral Vector Boost Vaccines

Pox viral vectors are developed for novel HIV-1 vaccine applications because of the rich knowledge accumulated from the success of using vaccines to eradicate smallpox globally and the availability of several well characterized recombinant pox vectors. Of note, the RV144 clinical trial, which achieved 31% efficacy against HIV-1 infection, used a canary pox vector as the priming vaccine.

One important pox vector platform is the modified vaccine virus Ankara (MVA). Researchers from Oxford University (Oxford, UK) used a DNA vaccine, composed of several T cell epitopes for HIV-1 including a fragment encoding the gag antigen as priming, followed by a MVA vaccine boost expressing the matched antigen. However, only low level T cell responses were generated, which may be due to limitations of epitope vaccines delivered by DNA vaccines [71,72,73,74,75,76]. 

The HVTN065 trial later tested a DNA vaccine containing several HIV-1 antigens as the priming immunization, followed by boost with a recombinant MVA expressing HIV-1 antigens. This DNA prime-MVA boost vaccine was well tolerated and produced detectable and reproducible HIV-1-specific cellular immunity in humans [77,78]. In this regimen, DNA priming was responsible for the induction of HIV-1-specific T cell immune response following the boost of MVA. The T cell responses were polyfunctional; about 50% of the HIV-specific CD4^+^ and CD8^+^ T cells induced in vaccinated subjects produced more than three cytokines. Furthermore strong T cell proliferation, as well as robust production of the T cell growth factor IL-2 by HIV-1 specific CD4^+^ and CD8^+^ cells was observed. A phase IIs clinical trial HVTN205 using a similar DNA prime-MVA boost regimen has been completed and the immune responses are currently under analysis. 

In addition to MVA, extensive research also went into NYVAC, a highly attenuated vaccinia virus vector. Results from the EuroVacc 02 trial using DNA prime-NYVAC boost demonstrated the safety and high immunogenicity of this platform [79]. The DNA and the NYVAC both expressed fused Gag-Pol-Nef and gp120 Env subunit of Clade C isolate, CN54. CD4^+^ T cell responses were detected in 90% of DNA prime-NYVAC boost vaccinees, which was superior to responses induced by NYVAC alone (33% of responders). However, the T cell responses were predominantly mediated by CD4 T cells, while only a low magnitude of CD8^+^ T cells specific for HIV-1 antigens Gag, Pol, and Nef were detected.

Clinical trials were also conducted using a HIV-1 DNA vaccine prime followed by boost with another pox vector, the recombinant fowlpox viral vector. In these studies, DNA vaccines pHIS-HIV-B or pHIS-AE encoding gag, pol, env, tat, vup and rev were delivered as the priming vaccine followed by a fowlpox vaccine boost (rFPV-HIV-M3) expressing gag and pol. This regimen was not effective in inducing positive HIV-1-specific T cell response in humans, despite the high immunogenicity revealed in pre-clinical study in a non-human primate model [80,81,82].

It is not clear why MVA or NYVAC is more effective than a fowlpox vector in the above studies. Furthermore, additional pox vectors based on less attenuated vaccinia viruses have been tested either alone or in combination with a DNA vaccine prime [83,84,85,86,87]. They showed promising immunogenicity results in animal studies including NHP models. Tiantan pox vector, developed by the China CDC, has been tested in humans as part of an HIV-1 DNA vaccine prime-pox vector boost regimen but data are not yet available. There is no comparative study to determine similarities and differences among different pox viral vectors and more importantly, how each vector system can further expand HIV-1-specific immune responses in hosts primed with HIV-1 DNA vaccines.

### DNA Prime—Protein Boost for Balanced Immune Responses Including Env-Specific Antibody Responses

Based on the early observation that HIV-1 DNA vaccines not only elicited T cell responses but also induced antigen-specific antibody responses [2,3], several studies further tested the idea of using DNA prime-protein boost immunization to further improve the level of HIV-1 Env-specific antibody responses in mice, guinea pigs, and even, rhesus monkeys [88,89,90]. While these early studies in the mid-1990s established the feasibility of combining DNA and recombinant protein vaccines in one immunization regimen, the true potential and exact mechanism of DNA priming to induce high quality Env-specific antibody responses were not yet fully appreciated; even after additional extensive research in the last decade, we have only just started to realize how much DNA immunization can contribute to the development of an effective AIDS vaccine in the context of a DNA prime-protein boost strategy.

Among a few DNA prime-protein boost studies conducted in healthy human volunteers, the “DP6-001” study provided the most comprehensive and promising immunogenicity data [91]. In this study, participants received three times priming immunizations with a polyvalent HIV-1 DNA vaccine, including six DNA plasmids (five expressing different primary gp120 antigens from clades A, B, C, and E and one expressing a clade C Gag antigen), followed by two times boost with a polyvalent recombinant gp120 protein vaccine (five primary gp120 antigens matching those used in the DNA prime). Although the DNA vaccine components were administered via intramuscular or intradermal needle injection without using an adjuvant or a physical delivery device (such as gene gun or EP), positive gp120-specific CD4^+^ T cell responses were detected in all volunteers at the end of three DNA immunizations, and were further boosted by the gp120 protein boost. It was also important to note that these effector memory CD4^+^ T cells were multifunctional, secreting IFN-γ, IL-2, and TNF-α. The subpopulation positive for CD154 maintained proliferative potential and could rapidly develop into mature effector CD4^+^ T cells [92]. Interestingly, this phenotype was also seen in long term nonprogressors or aviremic HIV-1 infected patients on highly active antiretroviral therapy (HAART). In the higher dose group (1.2 mg for Gag-expressing DNA vaccine component), positive Gag-specific CD4^+^ T cell responses were also detected at the end of three DNA immunizations, a response that was rare in previous DNA vaccine studies when no adjuvant or EP device was used. Antigen-specific CD8^+^ T cells were also observed in vaccinated participants [92].

However, the most significant finding was that high-titer gp120-specific antibody responses (end titration titers of 1:10^5^, equivalent to Env-specific antibody titers in chronically infected HIV-1 positive patients) were detected in immune sera of 100% of the DP6-001 trial participants following one or two gp120 protein boosts [91]. All of these immune sera had a broad reactivity recognizing gp120 antigens from HIV-1 subtypes A to E. High level neutralizing activities were easily detected against pseudotyped viruses expressing Env from the sensitive viruses (TCLA isolates and SF162), activities that were better than observed in a previously reported DNA prime-Ad5 vector boost vaccine trial [93], in which no neutralizing activities were detected even against these sensitive viruses. There are good levels of ADCC activities in vaccinees’ sera [94]. When DP6-001 trial volunteer immune sera were tested against the more difficult to neutralize pseudotyped viruses from clades A to E and positive NAb was determined as greater than 50% inhibition, sera from approximately 1/3 of the volunteers were able to neutralize 80%–100% of this pseudotyped virus panel; the other 1/3 of the immune sera was able to neutralize 50%–79% tested viruses, and the remaining 1/3 of the immune sera neutralized 25%–49% of the tested viruses; serum from only one volunteer could neutralize only one pseudotyped virus [91]. In addition, a neutralization assay was conducted against the most difficult to neutralize Tier 2 viruses, but the neutralizing activities were very low. In summary, the DP6-001 trial raised high level binding antibody and broad neutralizing antibody responses. Additional assays have been done to show high level anti-V2 antibodies in DP6-001 volunteers [45], similar to RV144 trial results. 

The DP6-001 trial also revealed a rare event with high level skin reactogenicity among volunteers including possible skin vasculitis, particularly after the protein boost [95]. This is very different from other DNA vaccine studies where the overall safety profile has been excellent. One possible reason is the use of a strong adjuvant, QS-21, in the protein boost; QS-21 is well-known for its potential in eliciting various adverse events. Future studies with other adjuvants with improved safety profiles are needed in DNA prime-protein boost studies.

The finding of high titer and high quality antibody responses in the DP6-001 trial was supported by another phase I DNA prime—Env protein boost clinical trial. A two-valent DNA vaccine prime, including one expressing an HIV-1 Gag and one expressing a V2-deleted gp140, were formulated in polylactice-coglycolides and delivered by intramuscular needle injection, followed by boost immunization using the recombinant gp140 protein matching that used in DNA prime. Compared to volunteers who received gp140 protein alone immunizations, DNA priming generated skewed Th1 phenotype polyfunctional Env-specific CD4^+^ T cells, a higher frequency of Env-specific memory B cells, and a higher titer of neutralizing antibodies and ADCC [96]. However, the breadth of neutralizing activities in this trial was limited, presumably due to limited valency of Env immunogens included in the formulation and/or a potential negative impact of V2 deletion to the quality of antibody responses.

Following the antibody results from DP6-001 trial, additional *in vitro* and *in vivo* studies further revealed the role of DNA prime immunization in generating high quality antibody responses.

In a rabbit study using the same formulation and immunization schedule as DP6-001, it was observed that rabbit immune sera elicited by recombinant gp120 protein alone mainly recognized the V3 eptiope while rabbit immune sera from the DNA-primed group exhibited unique binding against six additional gp120 epitopes, half of which contain residues that either are part of the CD4 binding site (CD4bs) or are involved in the binding with the CD4bs targeting neutralizing monoclonal antibody (mAb), b12 [97]. This finding indicated that DNA delivery of the gp120 immunogen is more effective than recombinant gp120 protein in eliciting conformation-sensitive epitopes. 

This finding was further validated by a comparative analysis with sera samples from three human HIV-1 vaccine studies: HVTN041 (recombinant gp120 protein alone vaccine formulated with potent AS02A adjuvant), HVTN203 (canarypox vector prime-gp120 protein boost), and DP6-001 study as discussed above (DNA prime-gp120 protein boost) [94]. Of note, HVTN203 is an early phase clinical study that used the same immunization regimen as the RV144 efficacy trial. HVTN041 sera had the highest binding antibody responses against the linear V3 epitope and high neutralizing activities against sensitive viruses but with limited breadth against other pseudotyped viruses. HVTN203 sera had lower V3 titers but otherwise a similar profile as the HVTN041 sera. DP6-001 sera demonstrated a higher frequency of positive neutralizing activities against more resistant viral isolates and a much higher frequency of CD4bs-specific antibody responses compared to HVTN041 and HVTN203 sera [94]. 

While the exact mechanism of how DNA priming is able to elicit CD4bs type antibody responses in both rabbit and human studies is unclear, it is possible that *in vivo* expression of HIV-1 envelope glycoprotein immunogens by DNA vaccination may have an advantage of maintaining the conformation of this sensitive protein compared to recombinant Env proteins manufactured by the traditional *in vitro* production and purification process. However, more recent small animal study results further indicate that DNA priming may also contribute to a more complicated process involving B cell development and innate immune mechanisms. It was demonstrated that DNA priming immunization was responsible for the improvement of the avidity of gp120-specific antibodies in rabbit immune sera [94]. The magnitude and profile of serum cytokines following recombinant gp120 protein immunization are changed with the addition of a priming step with gp120-expressing DNA vaccine [98].

The “Combination DNA and protein HIV vaccine” as it was called several years ago [99] may have other alternate regimens. For example: can the process be reversed by using a protein prime-DNA boost regimen to achieve the same enhanced immune responses? Furthermore, the idea of delivering DNA and protein at the same time has been tested in multiple studies. In an early mouse model, it was shown that adding a DNA vaccine component may actually inhibit cytokine responses elicited by protein vaccines [100,101,102,103,104]. However, as shown by three more recent animal study reports, the combination of DNA and protein vaccines, when delivered at the same time, was also more immunogenic than each component used alone [105,106,107]. This finding raised a number of interesting theoretical and practical questions. First, in this regimen, multiple immunizations were given, therefore, the DNA delivered at the early immunizations actually served as priming for the immune system, which was later boosted by the protein vaccine component. Therefore, in some way, this is a staggered DNA prime-protein boost vaccination regimen. However, since protein was also given during the first and later immunizations, one issue that needs to be determined is whether any priming effects by the protein vaccine may interfere with DNA priming. Second, the DNA vaccine may have a dual role as it can serve as an adjuvant through innate immunity pathways also in addition to its role of delivering a subunit antigen. Therefore, it needs to be determined if co-delivery of the DNA and protein vaccines results in an adjuvant effect for the DNA or if it acts as an antigen delivery tool or both. Finally, recent papers did not provide convincing evidence that co-delivery is better than sequential delivery for DNA and protein vaccines. One study design flaw is the use of larger total DNA and protein doses for co-delivery when compared to sequential delivery. Furthermore, co-delivery actually increases the number of immunizations for either DNA or protein components when compared to sequential immunizations, and thus, is not surprising to see improved immunogenicity. More studies in this area with better designs will provide useful guidance to develop the most effective approach for taking advantage of the unique features of both DNA and protein vaccines.

## 4. Conclusions

After 20 years’ effort, HIV-1 DNA vaccines have been systemically optimized from vector and antigen design to the inclusion of molecular adjuvants. However, their true utility in human studies was confirmed by two key events: (1) the use of DNA vaccine to prime the host immune system, especially for the induction of high titer and high quality Env-specific antibody responses, and (2) the availability and experience of using physical delivery approaches such as an EP device in humans to enhance the delivery and immunogenicity of DNA vaccines, which proved more effective for T cell immune responses.

The role of DNA vaccines as a priming immunization will be compared with the use of viral vectors. Results from the RV144 trial may validate the use of a pox vector to prime the host, but also opens the door for priming with DNA vaccines. The setback with Ad5 vectors reminded us of the potential unrealized risk of using viral vectors. Table 1 compares the differences between DNA and viral vector vaccines. The relative immunogenicity of DNA vaccines may be lower than viral vector vaccines but the expression of additional antigens from viral vector vaccines may cause complications from pre-existing immunity against these antigens and also prevent repeated use of viral vectors. Immune responses elicited by DNA vaccines are highly focused and can be boosted further by repeated immunizations. DNA vaccines have been proven safe in repeated human studies and it is easy to manufacture and transport DNA vaccines, important features for global applications. While the DP6-001 trial showed good cross-clade neutralizing antibodies, high level and broadly cross-reacting binding antibodies, including ADCC, were also identified in the volunteers [91,94], as recently shown in RV144 trial volunteers. Future clinical studies will shed light on the relative merit between these two leading prime-boost HIV vaccine strategies.

The use of EP revived the enthusiasm of DNA vaccination in humans but more questions need to be answered. The safety, public acceptability, cost, and finally, the magnitude of improved immunogenicity require further study. Furthermore, the incorporation of molecular adjuvants in the EP delivery approach also needs to be addressed.

**Table 1:** Features of DNA vaccines and viral vector-based vaccines.

	DNA	Viral vector
Potency of immunogenicity when used alone	Low	Low to moderate
Immune focus	High: Product of insert gene	Diffused: Products of both inserted and vector genes
Pre-existing immunity	None	May present
Repeated immunization	Possible	Less likely
Safety profile	High	Unknown to potential concern
Manufacturing	Easy	More complicated
